# Advances in the development of new vaccines for tuberculosis and Brazil’s role in the effort forward the end TB strategy

**DOI:** 10.1590/0074-02760240093

**Published:** 2024-10-04

**Authors:** Ana Paula Junqueira-Kipnis, Luciana Cesar de Cerqueira Leite, Júlio Croda, Erica Chimara, Anna Cristina C Carvalho, Ricardo Alexandre Arcêncio

**Affiliations:** 1 Universidade Federal de Goiás, Instituto de Patologia Tropical e Saúde Pública, Rede Goiana de Pesquisa em Tuberculose, Goiânia, GO, Brasil; 2 Instituto Butantan, Laboratório de Desenvolvimento de Vacinas, São Paulo, SP, Brasil; 3 Universidade Federal do Mato Grosso do Sul, Faculdade de Medicina, Mato Grosso do Sul, MS, Brasil; 4 Fundação Oswaldo Cruz-Fiocruz, Mato Grosso do Sul, MS, Brasil; 5 Yale School of Public Health, New Haven, CT, USA; 6 Instituto Adolfo Lutz, Núcleo de Tuberculose e Micobacterioses, São Paulo, SP, Brasil; 7 Fundação Oswaldo Cruz-Fiocruz, Instituto Oswaldo Cruz, Laboratório de Inovações em Terapias, Ensino e Bioprodutos, Rio de Janeiro, RJ, Brasil; 8 Universidade de São Paulo, Escola de Enfermagem de Ribeirão Preto, Ribeirão Preto, SP, Brasil; 9 Rede Brasileira de Pesquisas em Tuberculose - REDE TB, Parque Tecnológico da Universidade Federal do Rio de Janeiro, Rio de Janeiro, RJ, Brasil

**Keywords:** tuberculosis, vaccine, BCG, TB vaccine pipeline

## Abstract

Tuberculosis (TB) continues to be the world’s leading killer of infectious diseases. Despite global efforts to gradually reduce the number of annual deaths and the incidence of this disease, the coronavirus disease 19 (COVID-19) pandemic caused decreased in TB detection and affected the prompt treatment TB which led to a setback to the 2019 rates. However, the development and testing of new TB vaccines has not stopped and now presents the possibility of implanting in the next five years a new vaccine that is affordable and might be used in the various key vulnerable populations affected by TB. Then, this assay aimed to discuss the main vaccines developed against TB that shortly could be selected and used worldwide, and additionally, evidence the Brazilian potential candidates’ vaccines in developing in Brazil that could be considered among those in level advanced to TB end.

Tuberculosis (TB) remains a persistent global health threat that kills more than one million people every year (1.3 million in 2022), whereas a potential vaccine against the disease could save 8.5 million lives in the next decades.^1^ Although the Bacille Calmette-Guerin (BCG) vaccine has been available for almost 100 years, it fails to provide long-term protective immunity which contributes to the dramatic situation of TB across the world.^2^ After almost three decades of new TB vaccines development, several vaccines are currently in clinical trials. Additionally, the research data accumulated thru those decades culminated with a World Health Organization (WHO) initiative producing a guide for the development of vaccines against TB, prioritizing vaccines for prevention of pulmonary TB in adolescents and adults, and new vaccines with improved safety and efficacy with respect to BCG when administered in neonates and infants [Preferred product characteristics (PPC) document; WHO].^3^

*The BCG vaccine* - The BCG vaccine is the only vaccine currently licensed for the prevention of TB in humans and is also the oldest vaccine still in use in the world. With more than 100 years of use, the BCG vaccine (*in vitro* attenuated vaccine form of *Mycobacterium bovis*), in its different lineages, has shown to be effective in preventing severe forms of TB (tuberculous meningitis and miliary TB) when applied intradermally in neonates.^4^ ^,^ ^1^ Single-dose BCG vaccination for newborns is recommended by the WHO in high-burden TB countries, while in countries with a low burden of the disease, the vaccination recommendation is indicated in risk groups.^1^

As a live mycobacterium attenuated by periodic passages in culture media, BCG underwent mutations from the original BCG strain isolated by Calmett and Guérin at the Pasteur Institute, resulting in phenotypic and genotypic different BCG strains. Currently, nine different strains of BCG are produced in the world and this diversity raises questions about the different immunogenic capabilities of the strains and their potential side effects.^4^ There is no laboratory or clinical test able to effectively prove that an individual vaccinated with BCG is protected against *Mycobacterium tuberculosis* (Mtb) infection/disease. The use of tuberculin skin test (TST) conversion as an indication of proper immunization and protection has been shown to be inappropriate in clinical studies.^5^ Therefore, in the absence of an appropriate marker of the efficacy/effectiveness of BCG vaccination, BCG protection is based on data from randomized clinical trials and meta-analyses.

The protective effect of the BCG vaccine is higher when inoculated in individuals not infected by Mtb or not previously sensitized by environmental mycobacteria. The protection provided by BCG to prevent pulmonary TB can be very variable (from 0% to 80%). Age at BCG vaccination, sex, risk of TB in the study population, in addition to the prevalence of non-tuberculous mycobacteria in the region (which may vary with latitude, being lower the further away from the Equator line) are among the possible factors involved in the variation of the protection conferred by BCG.^4^ ^,^ ^5^ ^,^ ^6^ In the meta-analysis carried out by Colditz et al.,^5^ evaluating 1264 studies, vaccination with BCG reduced the risk of TB by 50% and death by 71%. However, in another systematic review, this protection reached 90% against tuberculous meningitis and miliary TB in infants.^6^ In a more recent meta-analysis of primary data, involving 26 cohorts from 17 different countries, the findings confirmed the protection of the BCG vaccine in children, particularly those under five years of age, but it was ineffective in preventing TB among adolescents and adults.^7^

As presented above, BCG vaccine provides questionable early protection against TB and adjunctly contributes to ameliorate the immune responses against other diseases by improving the immune responses to infants’ vaccines in a mechanism known as trained innate immune response.^8^ In this sense, evaluating if BCG revaccination could avoid TB in adults is reasonable. The BCG REVAC study evaluated the effect of BCG revaccination in a cluster-randomized cohort of school-aged children in 767 schools from two cities in Brazil (Salvador and Manaus), with a total of ~ 348.000 children. The efficacy of BCG revaccination on the incidence of TB as determined by the Brazilian Tuberculosis Control Program was 9%.^8^ A case-control study was performed in Recife in neighborhood-matched controls of TB cases showing an adjusted vaccine efficacy (VE) of ~ 8%.^9^ Latter reanalysis of the BCG REVAC data showed that VE of neonatal BCG immunization was comparable (~ 36-40%) in both cities, but first vaccination in school-aged children was high only in Salvador (farther from the equator), suggesting a blocking effect.^10^ Again, protection in revaccinated children was low or absent in both cities. On the other hand, a prevention of infection (POI) trial performed in South Africa, reported a 45.3 % reduction in sustained QTF conversion in ~ 2000 participants in BCG revaccinated individuals.^11^ The contradictory results on the role of BCG revaccination led the WHO to suspend the recommendation for BCG revaccination in school-age children and adolescents, as had happened in Brazil in the 1990s.^12^ ^,^ ^10^ Whether the BCG vaccine can prevent Mtb infection (and not just active forms of TB) is still another matter of controversy.^13^ ^,^ ^14^ ^,^ ^15^ ^,^ ^16^ ^,^ ^17^

*New TB vaccines in the pipeline* - Evolutionarily, Mtb acquired several evasion mechanisms against the host immune responses, thus developing new vaccines against TB that avoid Mtb infection (it is estimated that one quarter of the world population is infected) and prevent the disease is challenging.^1^ Nowadays, there are 22 vaccines cataloged at the vaccines pipelines (TBVI, Pipeline of vaccines - TBVI Pipeline; TBVI, Stop TB Partnership). Five vaccines are in phase 3, four in phase 2b and most of the other vaccines are being tested for safety and immunogenicity or in pre-clinical stages. Here, it will be presented the vaccines that are on phases 3 and 2b of clinical trials, and some vaccines that although are listed as phase 2a in the pipelines already initiated the phase 2b of clinical trial and the vaccines that were developed by Brazilian researchers (Table).

**TABLE:** Tuberculosis (TB) vaccine candidates and vaccines developed in Brazil

Candidate vaccine	Technology	Phase(s) of development	Target population
*Mycobacterium leprae* HSP65	DNA	Preclinical	
DNA-Ag85	DNA	Preclinical	
BCG ZMP1	Live	Preclinical	All
rBCG-LTAK63	Live	Preclinical	
H107	Protein subunit	Preclinical	Adolescents and adults
CysVac2/Ad	Protein subunit	Preclinical	Adolescents and adults
CMX-ADVAX-4	Protein subunit	Preclinical	
BCG-CMX	Protein subunit	Preclinical	
mc2-CMX	Protein subunit	Preclinical	
ChAdOx/MVA PPE15-85A (BCG Prime)	Viral vectored	Preclinical	Adolescents and adults
FNBPA+ pValac:e6ag85a	Live	Preclinical	
BNT164a1/BNT164b1	mRNA	1	Children, Adolescents and adults
AEC/BC02	Protein subunit	1	Adolescents and adults
Ad5 Ag85A	Viral vectored	1	Adolescents and adults
ChAdOx1.85A MVA85A	Viral vectored	1	Adolescents and adults
TB/Flu04L	Viral vectored	1	Adolescents and adults
MTBVAC	Live	2a	Adolescents and adults
ID93/GLA-SE	Protein subunit	2a/2b, 2b/3	Adolescents and adults
M72+ASO1E	Protein subunit	2b	Adolescents and adults
DAR-901	Whole cell	2b	Adolescents and adults
MTBVAC	Live	3	Infants and neonates
VPM1002	Live	3	All
GamTBvac	Protein subunit	3	Adolescents and adults
MIP immunovac	Whole cell	3	Adolescents and adults

Note: the vaccines were presented by the TBVI and StopTB partnership pipelines or literature search.

One of the strategies adopted was to use live attenuated vectors to carry Mtb antigens and induce protective, and long-lasting immune responses against TB. VPM1002 is a genetically modified BCG vaccine that induces improved Mtb specific immune responses compared to BCG. This vaccine does not have urease C, an important molecule used by mycobacteria to avoid lysosome and phagosome fusion by neutralizing the pH of the phagosome and consequently the bacterial clearance. Additionally, this BCG vaccine expresses listeriolysin, an enzyme that induces pore formation in the endosomal membrane facilitating mycobacterial presentation to both CD4+ and CD8+ T cells.^18^ ^,^ ^19^ Infant and neonatal clinical trials to evaluate VPM1002 safety and immunogenicity were performed in non-endemic and endemic countries, and VPM1002 vaccine was shown to induce a specific immune response against TB. However, in one of the studies, the immune response induced by VPM1002 was not superior to that induced by BCG. It is important to note that during this clinical trial, the BCG vaccine used as control had to be modified due to a shortage in supply.^19^ ^,^ ^20^ ^,^ ^21^ The immunogenicity of BCG vaccine strains is variable and thus this fact may have impacted the results. An open-labeled clinical trial that compared the efficacy of the VPM1002 with the BCG vaccine showed no difference. However, VPM1002 showed less severe adverse reactions at the injection site in newborn infants exposed to human immunodeficiency virus (HIV) when compared to BCG. The efficacy, safety, and immunogenicity conferred by VPM1002 are being evaluated in a multi-country phase III clinical trial that will be completed in 2025 (Phase III clinical trial: NCT04351685).

MTBVAC is a live attenuated Mtb vaccine that complies with the GENEVA and WHO safety requirements, presenting two deletions at known Mtb virulence genes. This vaccine presents deletions in PhoP, which is part of the two-component system PhoP/PhoR, and fadD26, that are essential for Mtb virulence. For instance, the PhoP/PhoR system is involved in the production of several immune evasion mechanisms, which after the deletion resulted in reduced production of molecules involved in lipid metabolism and cell wall constitution (*i.e.*, Ag85 secretion). Additionally, this deletion avoids the secretion of ESAT-6, a protein directly involved in immune evasion. On the other hand, fadD26 is crucial to the biosynthesis and exportation of phthiocerol dimycocerosates (PDIM), the main virulence-associated cell-wall lipids of Mtb.^22^ ^,^ ^23^ To comply with WHO and Geneva requirements for a life attenuated vaccine, MTBVAC was improved by the deletion of the antibiotic resistance gene used to select the vaccinal strains.^22^ Pre-clinical, phase I and phase II clinical trials showed that this vaccine induced response to a great variety of Mtb immunodominant antigens that were absent in the BCG vaccine.^24^ ^,^ ^25^ The efficacy, safety, and immunogenicity of MTBVAC are being evaluated at a randomized, double-blind clinical trial in newborns exposed or non-exposed to HIV in TB-endemic regions (Phase III clinical trial NCT04975178).

Protein-based vaccines are a different approach that have advantages in production scalability, biosafety, and reproducibility. Another advantage of using a protein-based vaccine compared to Mtb - based vaccine is the non-induction of immune response against antigens present in PPD or IGRA, preserving these diagnostic tests for TB infection. M72 is a hybrid polyprotein composed of two Mtb proteins known as Mtb32 and Mtb39. Combining the Mtb32 carboxy-terminal with Mtb39 and ending with Mtb32 amino terminal generated a protein with 72 kDa. The vaccine adjuvant AS01E (GlaxoSmithKline) is composed of liposome-based vaccine adjuvant system containing two immunostimulants: 3-O-desacyl-4’-monophosphoryl lipid A (MPL) derived from gram-negative bacteria lipopolysaccharide (LPS) that binds to TLR-4 present in the cell membrane and the saponin QS-21, derived from *Quillaja saponaria*, Molina tree, that destabilize the cellular plasmatic membrane.^26^ ^,^ ^27^ M72/AS01E has shown strong immune response in adults treated for TB, regardless of their HIV status and antiretroviral therapy.^28^ ^,^ ^29^ ^,^ ^30^ ^,^ ^31^ M72/AS01E generated strong antibody and polyfunctional M72-specific CD4 T cells, with the highest CD4 T cell responses observed in tuberculin skin test-negative adults. In infants, M72/AS01E vaccine showed a generally tolerable safety profile,^32^ regardless of whether it was given after or concurrently with other vaccines.^33^ However, the vaccine group experienced higher incidences of adverse events compared to the control group. These included redness at the injection site, headache, malaise, myalgia, fatigue, pain, swelling and fever.^30^

The M72/AS01E TB vaccine has shown promising efficacy results in clinical studies in endemic countries. Results of the final analysis of a Phase 2b trial of the M72/AS01E vaccine showed efficacy at month 36 of 49.7% to prevent active pulmonary TB.^31^ This result, from a three-year follow-up, gave hope for a vaccine that could be used to avoid development of active TB. Another large ongoing phase III clinical trial is enrolling adolescent and adult volunteers for a randomized, double-blinded study to assess the efficacy of a vaccine in preventing active TB. The trial aims to evaluate the vaccine’s potential in reducing TB development including those who are IGRA positive, negative, and living with HIV (Phase III clinical trial NCT06062238).

The GamTBvac is a recombinant protein vaccine comprising three antigens from Mtb in fusion with the dextran-binding domain (DBD) of *Leuconostoc mesenteroides*: DBD-Ag85A and DBD-ESAT6-CFP10. These fusion proteins are immobilized on dextran and mixed with an adjuvant consisting of DEAE-dextran core (a polycationic derivative of dextran), and the TLR9 agonist, CpG oligodeoxynucleotides. This constitutes a nanoparticle presentation of the recombinant subunit protein vaccine, including a combination of adjuvants, to be administered in two doses as a booster in BCG-vaccinated adolescents and adults (18-49 years). It is expected that the nanoparticle structure, together with the Th1-driving properties of the adjuvants, will increase the induction of cellular immune responses. Results in mice and guinea pigs showed that a prime-boost immunization with BCG: GamTBvac induced protection against Mtb challenge comparable to or slightly higher than BCG alone.^34^ The first human trial for GamTBvac (ClinicalTrials.gov ID NCT03255278) showed an acceptable safety profile and displayed immunogenicity against its antigens using a half dose (containing 12.5 µg of each fusion protein).^35^ ^,^ ^36^ These results led to a Phase 2 trial (ClinicalTrials.gov ID NCT03878004) that confirmed the safety and immunogenicity of the vaccine^35^ ^,^ ^36^ and strengthened the case for its entry into a Phase 3 trial (ClinicalTrials.gov ID NCT04975737).

The ID93+GLA-SE/QTP101 vaccine, currently in Phase 2a development, represents a significant advancement in the prevention of TB disease. This vaccine is a combination of the recombinant protein ID93 and the adjuvant GLA-SE (glycopiranosyl lipid adjuvant - stable emulsion). The ID93 protein itself comprises four Mtb antigens linked in tandem which contain antigens associated with Mtb virulence (RV1813, Rv2608, Rv3619, Rv3620).^32^ ^,^ ^37^ ^,^ ^38^ The GLA in its composition is a Toll-like receptor 4 agonist. It has been shown to induce an innate and adaptive immune response, mainly Th1 activation *in vivo* studies. Widely used in various experiments as part of other vaccines, it has resulted in effective protection in animal models.^39^ Preclinical studies reported protection against TB due to increased secretion of cytokines by Th1 CD4+ T lymphocytes. When used as a boost to a BCG prime, it has provided long-term protection against TB^40^ also the addition of a second TLR agonist, CpG DNA, has further improved the vaccine’s immunogenicity and protective efficacy in preclinical studies. The ID93+GLA-SE/QTP101 vaccine has shown promising results in clinical trials, with the GLA-SE adjuvant significantly enhancing immune responses.^37^ In addition, the safety of its composition and administered doses was verified, with a low incidence or absence of adverse events post-vaccination. These findings suggest that the ID93+GLA-SE/QTP101 vaccine holds promise as a potential TB vaccine candidate. The ID93+GLA is currently in phase IIa clinical trial where studies demonstrated its ability to induce specific humoral immune response and CD4^+^ T cells and a study with people living with HIV (PLWH) is in progress (ClinicalTrials.gov ID NCT02465216; NCT06205589).

DAR-901 is an inactivated whole cell vaccine derived from the non-tuberculous mycobacterium, *M. obuense.* The effectiveness of the DAR-901 TB vaccine as a booster to the BCG vaccine has produced varied findings in different studies. Munseri et al. discovered that it did not effectively prevent initial or persistent TB infection but did improve immune responses to specific antigens.^41^ On the contrary, Lahey et al. found that it stimulated cellular and humoral immunity and offered better protect compared to a homologous BCG boost.^42^ Both von Reyn et al. and Mosonou et al. concluded that the vaccine was safe and induced specific immune responses, although Mosonou et al. noted these responses were lower than those induced by BCG.^43^ ^,^ ^44^ Further investigation is necessary to comprehensively assess the efficacy of the DAR-901 vaccine when used as a booster for BCG.

Attenuated *Mycobacterium indicus prannii* vaccine, also known as MIP immunovac, has been evaluated for leprosy and TB for over 15 years. MIP immunovac has shown promise as a booster vaccine for TB, enhancing the immune response and providing higher protection in animal models of TB when given in combination with BCG.^45^ It has also been found to improve lung pathology and reduce bacterial burden when used in combination with standard chemotherapy for Mtb infection.^46^ This vaccine induces macrophage, Th1, and Th17 cell activation, regulates Th2 immune responses, and is safe when tested in leprosy and TB-endemic countries. This vaccine is now in a phase 3 clinical trial to evaluate the efficacy against TB in adults and adolescents in India (phase 3 clinical trial registered at Clinical Trial Registry -India: CTRI/2019/01/017026).

*Development of TB vaccines in Brazil* - The vaccines under development against TB in Brazil are all in the preclinical stage, but several strategies have been investigated. The first strategy that was extensively studied in Brazil started in the late 1990s and is based on immune stimulation by a DNA vaccine expressing *M. leprae* HSP65.^47^ Several delivery formulations and routes were investigated: DNA-HSP65 alone (i.m. or Gene Gun), loaded on PLGA microspheres with TDM adjuvant, DNA-HSP65 prime/microspheres loaded with rHsp65 as boost, BCG prime / DNA-HSP65 boost, with the use of different adjuvants such as CpG, TDM or KLK (11-mer antibacterial peptide (KLKL(5)KLK).^48^ The protective effect of this vaccine was shown to be comparable to BCG, with some increase in protection when used in prime-boost strategies in the mouse and guinea pig models.^49^ Furthermore, the DNA-HSP65 vaccine was evaluated as a therapeutic vaccine in combination with chemotherapy.^50^ Safety was recently confirmed in Cynomolgus monkeys.^51^

Antigen-specific DNA vaccines have also been investigated, first using DNA-Ag85. However, although Th1 immune response was increased, protection was observed only in the spleens.^52^ Expanding this strategy, a recombinant strain was derived, in which a *Lactococcus lactis* FNBPA+ was transformed with a DNA vaccine encoding either Ag85 alone or a fusion of ESAT-6 and Ag85A (pValac:e6ag85a) and delivered orally to mice. These strains induced significant increases in Th1/Th17 response and antigen-specific sIgA.^53^ Another DNA-Vaccine study was also based on antigen specific immunity: the DNA-APA (encoding the alanine proline antigen) was investigated in a BCG prime and boost with DNA-APA (in biodegradable microspheres), showing a 1.0 log CFU reduction in relation to BCG in mice, and a decrease in inflammation.^54^

Recombinant BCG (rBCG) is another strategy that has been explored by different groups in Brazil. Stable expression of Ag85B in rBCG was obtained by auxotrophic complementation.^55^ Immunization of cattle with this strain showed increased production of IL-17 upon PPD stimulation, correlating with improved histopathology scores in the lungs as compared with BCG immunization.^56^

Brazilian researchers also applied strategies to identify antigens that were recognized by individuals with TB, testing their antigenicity and eventually selecting immunodominant antigens and building chimeric proteins with higher immunogenicity and antigenicity. While the Ag85 complex (35% of the proteins in the cell wall) is recognized by the immune responses (IR) of individuals with active TB disease, HSP-X (alpha-crystallin) is recognized by IR of individuals infected with Mtb, but without clinical disease, and MPT-51 protein (that binds to fibronectin), is recognized by the IR of individuals with active and reactivated resistant TB.^57^ ^,^ ^58^ ^,^ ^59^ ^,^ ^60^ Thus, CMX protein was developed which is a chimeric protein composed of Ag85C, HSP-X, and MPT-51 immunodominant epitopes.^61^ The subunit vaccines composed of rCMX were evaluated with different adjuvants: CpG DNA, liposomes, or ADVAX-4,^62^ ^,^ ^63^ showing protection against TB challenge in preclinical assays. Additionally, rBCG expressing CMX or *M. smegmatis-*CMX administered with an rCMX boost induced higher protection against Mtb than BCG in mice.^64^ ^,^ ^65^ CMX was shown to be an TLR-4 agonist that induces Th1, Th17 and polyfunctional T lymphocytes.^64^ ^,^ ^65^ Additionally, CMX when evaluated as a diagnostic tool for TB, was shown to discriminate active TB from controls.^60^ ^,^ ^61^

Immune modulation of BCG was also investigated by expression of a strong Th1-driving adjuvant, the A subunit of the genetically detoxified Labile Toxin from *Escherichia coli*, generating rBCG-LTAK63. This strain induced higher protection against intratracheal or intranasal challenge with virulent Mtb strains, including a Beijing strain, as compared with BCG in mice.^66^ Furthermore, an immunoprotective effect was also observed in the lungs of immunized and challenged mice.^66^ ^,^ ^67^ A new strain without an antibiotic resistance gene (unmarked) was obtained through auxotrophic complementation.^68^ A lysine auxotroph was obtained by CRISPR/Cas, which was then complemented with a vector expressing the deleted gene and *ltak63*. This complemented auxotroph expresses LTAK63 without antibiotic resistance marker, presents stable maintenance of the plasmid, and induces long-term protection against Mtb challenge comparable to the original strain.^69^ This unmarked rBCG-LTAK63 strain presents characteristics considered suitable for use in human trials.

Two factors hinder the progress of the TB vaccine development into clinical trials in Brazil: the absence of preclinical toxicology testing (especially for class 2 live vaccines) and the limited availability of pilot GMP plants to obtain clinical lots of vaccines to be tested.

*Present and future perspectives* - We observed in the last years a substantial progress in TB vaccine development and currently, it is important to establish initiatives to accelerate their development. In January 2023, the WHO’s Director-General announced the creation of TB Vaccine Accelerator Council to facilitate the development, testing, authorization, and use of new TB vaccines. The Council is composed of Ministers of Health and Science, leaders of research and funding institutions, international organizations, and representatives from Civil Society. Currently, the chair of the Council is our Minister of Health, Dr Nisia Trindade, together with Indonesia’s Minister of Health, Dr Budi Gunadi Sadikin.

Another initiative towards promoting the development of new vaccines for TB is the organization of the 7th Global Forum on TB Vaccines, which will be held in Rio de Janeiro in October 2024. The main objectives of the Forum are: 1. to serve as a critical platform for advancing scientific knowledge, fostering collaboration, advocating for increased investment and political commitment, shaping policies, and 2. engaging with affected communities and their advocacy to accelerate progress towards TB vaccines.

The Brazilian participation in the Forum aims also to increase the visibility and recognition of the potential candidates for Brazilian TB vaccines. Unfortunately, in recent decades, Brazil has been losing its capacity in the development and production of vaccines. Lack of prioritization by health authorities and politics; high dependence on the international market, economic interests of large pharmaceutical companies; financial issues (low funding specifically for TB research), and the prioritization of other productive sectors over the health industrial complex may be among the factors to explain the fact that we are not at the forefront of developing new vaccines for TB. Collaboration with national and international partners and continued investment in research and development efforts may contribute to the progress on the main goal of a new TB vaccine in the coming years. These are the reasons initiatives such as Forum Global on TB vaccines are highly endorsed.

An issue that should be added to this discussion is the cost and context of developing a new vaccine. The incidence of TB in Brazil is intermediate when compared to other countries with also higher burden of TB. However, vulnerable populations such as people deprived of their liberty have a TB incidence rate equivalent to that of African countries that should be considered in clinical trials of TB vaccines. Addressing this issue poses a significant challenge, particularly from an ethical perspective, as ensuring voluntary participation and availability is a barrier mainly because the individuals are under State custody, which may have their free will to enroll compromised. In relation to costs, particularly during phases 2 or 3, the expenses associated with testing the vaccine surpass the threshold of $500 million USD, which poses a significant constraint, particularly for countries with limited financial resources. Despite these challenges, we remain enthusiastic about the prospect of having a new TB vaccine in the next few years. Regardless of its origin, our priority is to ensure access to all who need it, especially vulnerable populations.
